# Supplementary material for: Soy-Based Infant Formula is Associated with an Increased Prevalence of Comorbidities in Fragile X Syndrome
Source: Nutrients. 2020 Oct 14;12(10):3136. doi: 10.3390/nu12103136 (PMC7602206; doi:10.3390/nu12103136)
Supplement: Supplementary file 1 [file nutrients-12-03136-s001.pdf]

nutrients

Supplementary Data

Soy-Based Infant Formula is Associated with an Increased Prevalence of Comorbidities in Fragile X Syndrome

Cara J. Westmark [1]*, Chad Kniss [2], Emmanuel Sampene [3], Angel Wang [4], Amie Milunovich [5], Kelly Elver [2], David Hessl [6], Amy Talboy [7], Jonathon Picker [8], Barbara Haas-Givler [9], Amy Esler [10], Andrea L. Gropman [11], Ryan Uy [11], Craig Erickson [12], Milen Velinov [13], Nicole Tartaglia [14] and Elizabeth M. Berry-Kravis [4]

[1] Department of Neurology, University of Wisconsin, Madison, WI; westmark@wisc.edu
[2] Survey Center, University of Wisconsin, Madison, WI; ckniss@ssc.wisc.edu; kelver@ssc.wisc.edu
[3] Department of Biostatistics & Medical Informatics; University of Wisconsin, Madison, WI; sampene@biostat.wisc.edu
[4] Department of Pediatrics, Rush University Medical Center; Chicago, IL; angel_wang@rush.edu; elizabeth_berry-kravis@rush.edu
[5] National Fragile X Foundation, Folsom, CA; amie@fragileX.org
[6] MIND Institute and Department of Psychiatry and Behavioral Sciences, University of California, Davis, CA; drhessl@ucdavis.edu
[7] Departments of Human Genetics and Pediatrics, Emory University, Atlanta, GA; amy.talboy@emory.edu
[8] Boston Children's Hospital, Boston, MA; jonathon.picker@childrens.harvard.edu
[9] Autism & Developmental Medicine Institute, Geisinger Lewisburg, Lewisburg, PA; bahaasgivler@geisinger.edu
[10] Department of Pediatrics, University of Minnesota, Minneapolis, MN; else0007@umn.edu
[11] Children's National Health System, Washington, DC; AGropman@childrensnational.org; RSUY@childrensnational.org
[12] Cincinnati Children's Hospital Medical Center, Cincinnati, OH; craig.erickson@cchmc.org
[13] Institute for Basic Research in Developmental Disabilities, Staten Island, New York, milen.velinov@opwdd.ny.gov
[14] Department of Pediatrics, University of Colorado, Aurora, CO; Nicole.tartaglia@childrenscolorado.org
* Correspondence:westmark@wisc.edu; Tel.: 1+608-262-9730

Supplementary Table 1

Supplementary Table 1. Analysis of FXS comorbidities as a function of sex and soy-based infant formula.

Phenotype	Females Soy % (N)	Females No Soy % (N)	P^1	Males Soy % (N)	Males No Soy % (N)	P^1
none	20 (10)	42 (31)	0.28	3.8 (26)	30 (74)	0.0061
autism	50 (10)	19 (36)	0.052	69 (29)	53 (90)	0.14
food allergies	10 (10)	17 (35)	1.0	20 (30)	5.8 (86)	0.022
diabetes	0 (10)	2.7 (37)	1.0	0 (31)	1.1 (89)	1.0
GI problems	30 (10)	22 (36)	0.68	52 (33)	26 (93)	0.0068
seizures	20 (10)	7.7 (39)	0.27	23 (31)	13 (95)	0.18
allergies	70 (10)	41 (37)	0.10	52 (33)	31 (95)	0.030

[1] Chi-squared test was used unless any variable contained less than N=5 in which case Fisher exact test was used.

nutrients

Supplementary Data

Soy-Based Infant Formula is Associated with an Increased Prevalence of Comorbidities in Fragile X Syndrome

Cara J. Westmark [1]*, Chad Kniss [2], Emmanuel Sampene [3], Angel Wang [4], Amie Milunovich [5], Kelly Elver [2], David Hessl [6], Amy Talboy [7], Jonathon Picker [8], Barbara Haas-Givler [9], Amy Esler [10], Andrea L. Gropman [11], Ryan Uy [11], Craig Erickson [12], Milen Velinov [13], Nicole Tartaglia [14] and Elizabeth M. Berry-Kravis [4]

[1] Department of Neurology, University of Wisconsin, Madison, WI; westmark@wisc.edu
[2] Survey Center, University of Wisconsin, Madison, WI; ckniss@ssc.wisc.edu; kelver@ssc.wisc.edu
[3] Department of Biostatistics & Medical Informatics; University of Wisconsin, Madison, WI; sampene@biostat.wisc.edu
[4] Department of Pediatrics, Rush University Medical Center; Chicago, IL; angel_wang@rush.edu; elizabeth_berry-kravis@rush.edu
[5] National Fragile X Foundation, Folsom, CA; amie@fragileX.org
[6] MIND Institute and Department of Psychiatry and Behavioral Sciences, University of California, Davis, CA; drhessl@ucdavis.edu
[7] Departments of Human Genetics and Pediatrics, Emory University, Atlanta, GA; amy.talboy@emory.edu
[8] Boston Children's Hospital, Boston, MA; jonathon.picker@childrens.harvard.edu
[9] Autism & Developmental Medicine Institute, Geisinger Lewisburg, Lewisburg, PA; bahaasgivler@geisinger.edu
[10] Department of Pediatrics, University of Minnesota, Minneapolis, MN; else0007@umn.edu
[11] Children's National Health System, Washington, DC; AGropman@childrensnational.org; RSUY@childrensnational.org
[12] Cincinnati Children's Hospital Medical Center, Cincinnati, OH; craig.erickson@cchmc.org
[13] Institute for Basic Research in Developmental Disabilities, Staten Island, New York, milen.velinov@opwdd.ny.gov
[14] Department of Pediatrics, University of Colorado, Aurora, CO; Nicole.tartaglia@childrenscolorado.org
* Correspondence:westmark@wisc.edu; Tel.: 1+608-262-9730

Appendix A

RUSH UNIVERSITY
Children's Hospital

Fragile X-Associated Disorders Program
Elizabeth M. Berry-Kravis, MD, Ph.D.,
Fragile X Clinic
Deborah A. Hall MD, FXTAS Clinic
Mary Wood-Molo MD, FXPOI Clinic
1725 W. Harrison Street, Suite 718
CHICAGO, ILLINOIS 60612-3824
Tel. (312) 942-4036; Fax. (312) 942-4168

7/12/19

Dear Fragile X Caregiver,

Sometimes researchers want people with Fragile X to participate in research studies. These studies often help doctors and researchers find better ways to treat patients with Fragile X. You have the opportunity to participate in one of these studies.

Dr. Cara Westmark, a researcher from the University of Wisconsin-Madison, is looking for primary caregivers of persons with Fragile X to participate in a mail survey study. Your name was selected from a list of people enrolled in the Fragile X Online Registry with Accessible Research Dataset (FORWARD). Enclosed is information about Dr. Westmark's study and directions for what you need to do if you want to participate. **You don't have to take part in this study.** Your decision on whether to participate is up to you. Your participation in the FORWARD Registry & Database won't be affected in any way.

To participate in Dr. Westmark's study, please return your contact information on the enclosed card in the postage-paid envelope to the University of Wisconsin Survey Center (UWSC) as soon as possible. The UWSC will contact you directly by mail with a copy of the survey. A copy of their recruitment flyer is attached to this letter.

If you participate in this study, your answers to the survey questions are completely confidential and results of the study will only be shared in group form. All study forms will have a study number affixed, but none will contain your name or any other information that would allow you to be identified.

If you have any questions regarding the survey, you may contact Dr. Westmark at (608) 262-9730. If you have any questions about your rights as a research subject or have complaints about the research study or study team, contact UW Health Patient Relations at 608-263-8009. The Patient Relations Representatives work with research subjects to address concerns about research participation and assist in resolving problems.

Thank you for your time. We hope that many of you will be able to participate in this worthwhile study.

Sincerely,

Elizabeth Berry-Kravis MD, PhD
Professor of Pediatrics, Neurological Sciences
Director, Fragile X-Associated Disorders Program
Director, Fragile X Clinic and Research Program
Rush University Medical Center

In Support of:
Cara Westmark, PhD
Assistant Professor, Department of Neurology
University of Wisconsin Madison
Ph: (608) 262-9730
E: westmark@wisc.edu

Fragile X Syndrome Nutrition Study

OBJECTIVE

Researchers at the University of Wisconsin-Madison are inviting caregivers of persons with the full Fragile X mutation to take part in a new survey that studies the effects of early-life nutrition on disease outcomes

WHO CAN PARTICIPATE

- Primary caregivers of persons with the full Fragile X mutation
    - Fragile X persons may be male or female
    - Fragile X persons may be any age
- You may complete the survey for multiple Fragile X dependents
- Primary caregivers must already be participants in the FORWARD Database

THE STUDY INVOLVES

- Completion of a 10-page mail survey (55 questions) regarding your child's diet and behavior that will take approximately 20 min to complete
- You will receive a small monetary incentive

HOW CAN YOUR FAMILY BENEFIT

- There will be no direct benefit to you and your family, but your participation will further our understanding of Fragile X syndrome and may lead to new treatments

WHO IS FUNDING THIS STUDY

- This study is funded by a grant from the Eunice Kennedy Shriver National Institute of Child Health and Human Development (NICHD)
- The Principle Investigator is Dr. Cara Westmark at the University of Wisconsin-Madison

FOR MORE INFORMATION

- If you are not already a participant in the FORWARD Database and would like more information about this, please go to: http://forwardfx.org/collaborating-clinics/

Yes, I would like to participate in the

Fragile X Syndrome Nutrition Study

My contact information is:

Name ________________________________

Street 1 ______________________________

Street 2 ______________________________

City ______________________________

State ________ Zip ____________

Number Fragile X persons you care for: _____

Yes, I would like to participate in the

Fragile X Syndrome Nutrition Study

My contact information is:

Name ________________________________

Street 1 ______________________________

Street 2 ______________________________

City ______________________________

State ________ Zip ____________

Number Fragile X persons you care for: _____

Yes, I would like to participate in the

Fragile X Syndrome Nutrition Study

My contact information is:

Name ________________________________

Street 1 ______________________________

Street 2 ______________________________

City ______________________________

State ________ Zip ____________

Number Fragile X persons you care for: _____

Yes, I would like to participate in the

Fragile X Syndrome Nutrition Study

My contact information is:

Name ________________________________

Street 1 ______________________________

Street 2 ______________________________

City ______________________________

State ________ Zip ____________

Number Fragile X persons you care for: _____

University of Wisconsin
SCHOOL OF MEDICINE
AND PUBLIC HEALTH

Department of Neurology

<Date, 2019>

<Name>
<Address>
<City, State, Zip>

Dear <First Last>,

As a caregiver of a person with Fragile X, you play a vital role in medical research for the disorder. Thank you for providing your contact information indicating your willingness to participate in the **Fragile X Nutrition Study** at the University of Wisconsin-Madison, which is funded by the National Institute of Child Health and Human Development (NICHD). We are conducting this study to better understand how early-life nutrition affects Fragile X outcomes. This is important because dietary interventions that reduce symptoms could be beneficial to persons with Fragile X.

Enclosed you will find a brief questionnaire focusing on Fragile X. The survey should take about 20 minutes to complete. Your answers to these questions are completely confidential and results of the study will only be shared in group form. All study forms will have a study number affixed to them, but none will contain your name, the name of your child, or any other information that would identify you/them.

Your participation in this research study may benefit families with Fragile X in the future by helping us learn more about Fragile X. Your participation in this research study is voluntary. If you decide not to participate, your relationship with FORWARD Registry & Database will not be affected in any way. If you wish to opt out of future mailings for this study, please return a blank questionnaire in the envelope provided.

Protected health information (PHI) is information about your physical or mental health that includes your name or other information that can identify you. To do this study, we will use things you tell the research team about your child's health.

Your authorization for researchers to use this PHI does not have an end date. However, you can choose to take back your authorization for researchers to use your health information. You can do this at any time before or during your participation in the research. If you take back your authorization, you will not be able to take part in the research study. To take back your authorization, you will need to notify the research team by contacting Chad Kniss at the UW Survey Center at (608) 262-4157, or toll free at (800) 291-8624, extension 1388.

We hope that everyone we have contacted will respond in order to have the results be as accurate as possible. Please consider taking a few moments to complete and return the questionnaire in the postage-paid envelope provided. You can skip any survey questions that you do not want to answer. Even if you start the survey, you are not required to complete it. You can stop at any time

As a token of our appreciation for your assistance, please accept the enclosed $2 bill to spend on a little something for yourself, perhaps a cup of coffee or tea. We look forward to hearing from you.

If you have any questions regarding the survey, you may contact Chad Kniss at the UW Survey Center at (608) 262-4157, or toll free at (800) 291-8624, extension 1388. If you have any questions about your rights as a research subject or have complaints about the research study or study team, contact UW Health Patient Relations at 608-263-8009. The Patient Relations Representatives work with research subjects to address concerns about research participation and assist in resolving problems.

Thank you so much for your thoughts and your time. Your opinions and experiences are very important to us.

Sincerely,

Cara J. Westmark

Cara Westmark, PhD
Assistant Professor
Department of Neurology
University of Wisconsin-Madison
Ph: (608) 262-9730
E: westmark@wisc.edu

About a week ago, we mailed you a Fragile X questionnaire that included questions about nutrition. If you have already returned the survey, thank you for your participation. If not, **please take a moment to complete and return the survey today**. With your help, this research will enhance the ability of medical professionals to help families with Fragile X.

If you have questions about this research, please contact me at (608) 262-9730, or westmark@wisc.edu.

Thank you for your time and participation.

Sincerely,

Cara Westmark
Assistant Professor
University of Wisconsin

About a week ago, we mailed you a Fragile X questionnaire that included questions about nutrition. If you have already returned the survey, thank you for your participation. If not, **please take a moment to complete and return the survey today**. With your help, this research will enhance the ability of medical professionals to help families with Fragile X.

If you have questions about this research, please contact me at (608) 262-9730, or westmark@wisc.edu.

Thank you for your time and participation.

Sincerely,

Cara Westmark
Assistant Professor
University of Wisconsin

About a week ago, we mailed you a Fragile X questionnaire that included questions about nutrition. If you have already returned the survey, thank you for your participation. If not, **please take a moment to complete and return the survey today**. With your help, this research will enhance the ability of medical professionals to help families with Fragile X.

If you have questions about this research, please contact me at (608) 262-9730, or westmark@wisc.edu.

Thank you for your time and participation.

Sincerely,

Cara Westmark
Assistant Professor
University of Wisconsin

About a week ago, we mailed you a Fragile X questionnaire that included questions about nutrition. If you have already returned the survey, thank you for your participation. If not, **please take a moment to complete and return the survey today**. With your help, this research will enhance the ability of medical professionals to help families with Fragile X.

If you have questions about this research, please contact me at (608) 262-9730, or westmark@wisc.edu.

Thank you for your time and participation.

Sincerely,

Cara Westmark
Assistant Professor
University of Wisconsin

University of Wisconsin
SCHOOL OF MEDICINE
AND PUBLIC HEALTH

Department of Neurology

<Date, 2019>

<Name>
<Address>
<City, State, Zip>

Dear <First Last>,

A few weeks ago, we sent you a questionnaire about Fragile X. **I am writing to encourage you to fill out your questionnaire and return it as soon as you can. We have enclosed a copy of the questionnaire along with a postage-paid, self-addressed envelope for your convenience**. If you have already returned your completed questionnaire, please disregard this reminder—we thank you for your help.

The questionnaire should take about 20 minutes to complete. Your answers to these questions are completely confidential and results of the study will only be shared in group form. All study forms will have a study number affixed, but none will contain your name or any other information that would allow you to be identified.

Your participation in this research study may benefit other families with Fragile X in the future by helping us learn more about Fragile X. Your participation in this research study is voluntary. If you decide not to participate, your relationship with the FORWARD Registry & Database will not be affected in any way. If you wish to opt out of future mailings for this study, please return a blank questionnaire in the envelope provided.

If you have any questions regarding the survey, you may contact Chad Kniss at the UW Survey Center at (608) 262-4157, or toll free at (800) 291-8624, extension 1388. If you have any questions about your rights as a research subject or have complaints about the research study or study team, contact UW Health Patient Relations at 608-263-8009. The Patient Relations Representatives work with research subjects to address concerns about research participation and assist in resolving problems.

Thank you so much for your thoughts and your time. Your opinions and experiences are very important to us.

Sincerely,

Cara Westmark, PhD
Assistant Professor
Department of Neurology
University of Wisconsin-Madison
Ph: (608) 262-9730
E: westmark@wisc.edu

University of Wisconsin
SCHOOL OF MEDICINE AND PUBLIC HEALTH

Department of Neurology

<Date, 2019>

<Name>
<Address>
<City, State, Zip>

Dear <First Last>,

Recently I wrote asking for your help with an important research study about Fragile X. **I am writing one final time to encourage you to fill out your questionnaire and return it as soon as you can. We have enclosed a copy of the questionnaire along with a postage-paid, self-addressed envelope for your convenience**. If you have already returned your completed questionnaire, please disregard this reminder—we thank you for your help.

The questionnaire should take about 20 minutes to complete. Your answers to these questions are completely confidential and results of the study will only be shared in group form. All study forms will have a study number affixed, but none will contain your name or any other information that would allow you to be identified.

Your participation in this research study may benefit other families with Fragile X in the future by helping us learn more about Fragile X. Your participation in this research study is voluntary. If you decide not to participate, your relationship with the FORWARD Registry & Database will not be affected in any way.

If you have any questions regarding the survey, you may contact Chad Kniss at the UW Survey Center at (608) 262-4157, or toll free at (800) 291-8624, extension 1388. If you have any questions about your rights as a research subject or have complaints about the research study or study team, contact UW Health Patient Relations at 608-263-8009. The Patient Relations Representatives work with research subjects to address concerns about research participation and assist in resolving problems.

Thank you so much for your thoughts and your time. Your opinions and experiences are very important to us.

Sincerely,

Cara J. Westmark

Cara Westmark, PhD
Assistant Professor
Department of Neurology
University of Wisconsin-Madison
Ph: (608) 262-9730
E: westmark@wisc.edu

nutrients

Supplementary Data

Soy-Based Infant Formula is Associated with an Increased Prevalence of Comorbidities in Fragile X Syndrome

Cara J. Westmark [1*], Chad Kniss [2], Emmanuel Sampene [3], Angel Wang [4], Amie Milunovich [5], Kelly Elver [2], David Hessl [6], Amy Talboy [7], Jonathon Picker [8], Barbara Haas-Givler [9], Amy Esler [10], Andrea L. Gropman [11], Ryan Uy [11], Craig Erickson [12], Milen Velinov [13], Nicole Tartaglia [14] and Elizabeth M. Berry-Kravis [4]

[1] Department of Neurology, University of Wisconsin, Madison, WI; westmark@wisc.edu
[2] Survey Center, University of Wisconsin, Madison, WI; ckniss@ssc.wisc.edu; kelver@ssc.wisc.edu
[3] Department of Biostatistics & Medical Informatics; University of Wisconsin, Madison, WI; sampene@biostat.wisc.edu
[4] Department of Pediatrics, Rush University Medical Center; Chicago, IL; angel_wang@rush.edu; elizabeth_berry-kravis@rush.edu
[5] National Fragile X Foundation, Folsom, CA; amie@fragileX.org
[6] MIND Institute and Department of Psychiatry and Behavioral Sciences, University of California, Davis, CA; drhessl@ucdavis.edu
[7] Departments of Human Genetics and Pediatrics, Emory University, Atlanta, GA; amy.talboy@emory.edu
[8] Boston Children's Hospital, Boston, MA; jonathon.picker@childrens.harvard.edu
[9] Autism & Developmental Medicine Institute, Geisinger Lewisburg, Lewisburg, PA; bahaasgivler@geisinger.edu
[10] Department of Pediatrics, University of Minnesota, Minneapolis, MN; else0007@umn.edu
[11] Children's National Health System, Washington, DC; AGropman@childrensnational.org; RSUY@childrensnational.org
[12] Cincinnati Children's Hospital Medical Center, Cincinnati, OH; craig.erickson@cchmc.org
[13] Institute for Basic Research in Developmental Disabilities, Staten Island, New York, milen.velinov@opwdd.ny.gov
[14] Department of Pediatrics, University of Colorado, Aurora, CO; Nicole.tartaglia@childrenscolorado.org
* Correspondence:westmark@wisc.edu; Tel.: 1+608-262-9730

Appendix B

Fragile X Syndrome Nutrition Study

Please complete the questionnaire below thinking about your child with Fragile X Syndrome.

For consistency, the words "child" and "parent" are used throughout the questionnaire although we recognize that you may be reporting about a person who is now a teen or adult, and that you may be a relative or caretaker, rather than the parent of the child.

1. Does your child with Fragile X Syndrome also have…

	Yes	No	Don't know
**a.** …Autism?	○	○	○
**b.** …Down Syndrome?	○	○	○
**c.** …Epilepsy?	○	○	○
**d.** …food allergies?	○	○	○
**e.** …Diabetes?	○	○	○

2. Does your child with Fragile X Syndrome also have a history of gastrointestinal problems?

○ Yes
○ No → **Go to question 4**
○ Don't know → **Go to question 4**

3. How old was your child when these problems started?

[ ] Weeks / Months / Years ***(Please circle one.)***

4. Does your child with Fragile X Syndrome also have a history of seizures?

○ Yes
○ No → **Go to question 15 on page 3**
○ Don't know → **Go to question 15 on page 3**

5. The next questions ask about the type of seizures your child has had. If you do not recall if you were ever told they had that type of seizure, please check don't know. Have you been told your child with Fragile X Syndrome has ever had…

	Yes	No	Don't know
**a.** …febrile seizures (with fever)?	○	○	○
**b.** …atonic seizures (drop attacks)?	○	○	○
**c.** …generalized tonic clonic seizures (grand mal)?	○	○	○
**d.** …absence seizures (petit mal)?	○	○	○
**e.** …simple partial seizures (focal)?	○	○	○
**f.** …complex partials?	○	○	○
**g.** …infantile spasms?	○	○	○

6. **Does your child have a history of some <u>other</u> type of seizure? Please describe their other type of seizure.**

[ ]

7. **At what age did your child have his or her <u>first</u> seizure?**

[ ] Weeks / Months / Years ***(Please circle one.)***

8. **Sometimes children have periods where their seizures are more frequent. Was there a time when your child's seizures were more frequent than they had been previously, or than they have been since that time?**

○ Yes
○ No → **Go to question 11**
○ Don't know → **Go to question 11**

9. **At about what age were your child's seizures happening most often?**

[ ] Weeks / Months / Years ***(Please circle one.)***

10. **Thinking about the time when your child's seizures were happening most often, what was the average number of seizures your child had?**

[ ] Per day/ Per week / Per month / Per year ***(Please circle one.)***

11. **Was your child given medication to treat the seizures?**

○ Yes
○ No → **Go to question 14 on page 3**
○ Don't know → **Go to question 14 on page 3**

12. **Were multiple medications required to treat the seizures?**

○ Yes
○ No
○ Don't know

13. **Did the medication(s) reduce the seizures?**

○ Yes
○ No
○ Don't know

14. In what month, day and year did your child have his or her last seizure?

MM | DD | YYYY

15. Does your child with Fragile X Syndrome also have a history of allergies?

○ Yes
○ No → **Go to question 18**
○ Don't know → **Go to question 18**

16. How old was your child when he or she first began to experience allergies?

Weeks / Months / Years ***(Please circle one.)***

17. Some examples of allergens might include pollen, dust, pets, latex, eggs, fish, gluten or wheat, milk, nuts, medications, and many others.

What are the allergens your child reacts to now or has in the past?

18. For the next questions, please think back to your child's first year of life.

During the first year of life, was your child with Fragile X Syndrome fed any breastmilk?

○ Yes
○ No → **Go to question 23 on page 4**
○ Don't know → **Go to question 23 on page 4**

19. How old was your child when he or she began to be fed breastmilk?

Days/ Weeks / Months ***(Please circle one.)***

20. Some examples of why people might choose to feed their child breastmilk include doctor recommendations, family recommendations, or a belief that it is healthiest for the baby.

Why was your child fed breastmilk?

21. How old was your child when he or she stopped being fed breastmilk?

Days/ Weeks / Months ***(Please circle one.)***

22. Some examples of why people might choose to stop feeding their child breastmilk include being painful for the mother, or the baby not getting enough milk.

What was the reason your child stopped being fed breastmilk?

23. Was your child with Fragile X Syndrome fed any cow milk formula in his or her first year of life?

- ○ Yes
- ○ No → **Go to question 28 on page 5**
- ○ Don't know → **Go to question 28 on page 5**

24. How old was your child when he or she began to be fed cow milk formula?

[ ] Days/ Weeks ***(Please circle one.)***

25. Some examples of why people might choose to feed their child cow milk formula include doctor recommendations, family recommendations, or an inability to breastfeed.

Why was your child fed cow milk formula?

26. How old was your child when he or she stopped being fed cow milk formula?

[ ] Days/ Weeks / Months ***(Please circle one.)***

27. Some examples of why people might choose to stop feeding their child cow milk formula include allergy, intolerance, constipation, diarrhea, too much mucus, gas, too much spit up, vomiting, or parental choice.

What was the reason your child stopped being fed cow milk formula?

28. Was your child with Fragile X Syndrome fed any soy-based formula in his or her first year of life?

- ◯ Yes
- ◯ No → **Go to question 33**
- ◯ Don't know → **Go to question 33**

29. How old was your child when he or she began to be fed soy-based formula?

[ ] Days/ Weeks ***(Please circle one.)***

30. Some examples of why people might choose to feed their child soy-based formula include problems with other foods such as allergy, intolerance, constipation, diarrhea, too much mucus, gas, too much spit up, vomiting, or parental choice.

Why was your child fed soy-based formula?

[ ]

31. How old was your child when he or she stopped being fed soy-based formula?

[ ] Days/ Weeks / Months ***(Please circle one.)***

32. Some examples of why people might choose to stop feeding their child soy-based formula include allergy, intolerance, constipation, diarrhea, too much mucus, gas, too much spit up, vomiting, or parental choice.

What was the reason your child stopped being fed soy-based formula?

[ ]

33. Some examples of specialty formulas include amino acid, rice, or meat-based formulas. Was your child with Fragile X Syndrome fed any specialty formulas in his or her first year of life?

- ◯ Yes
- ◯ No → **Go to question 38 on page 6**
- ◯ Don't know → **Go to question 38 on page 6**

34. How old was your child when he or she began to be fed a specialty formula?

[ ] Days/ Weeks ***(Please circle one.)***

35. Some examples of why people might choose to feed their child specialty formulas include problems with other foods such as allergy, intolerance, constipation, diarrhea, too much mucus, gas, too much spit up, vomiting, or parental choice.

Why was your child fed other specialty formulas?

36. How old was your child when he or she stopped being fed a specialty formula?

☐ Days/ Weeks / Months ***(Please circle one.)***

37. Some examples of why people might choose to stop feeding their child other specialty formulas include allergy, intolerance, constipation, diarrhea, too much mucus, gas, too much spit up, vomiting, or parental choice.

What was the reason your child stopped being fed a specialty formula?

38. For the next questions, please think about the biological family of your child with Fragile X Syndrome.

Did anyone in the child's biological family including mother, father, brother, or sister have any of the following, and if yes, what was the relationship of that family member to the child?

**Condition**	**Don't know**	**No**	**Yes**		**Specify Relationship to Child**
Asthma	○	○	○	→	
Allergies	○	○	○	→	
Thyroid Condition	○	○	○	→	
Seizures	○	○	○	→	
Autoimmune Condition	○	○	○	→	
Juvenile onset Diabetes	○	○	○	→	
Adult onset Diabetes	○	○	○	→	

39. If you indicated a family member had an autoimmune condition, what was it?

40. For the next questions, we want you to try to remember how your child behaved when he or she was about 3 years old. If you were not with your child at that age, or you do not remember how they behaved, you may skip any one of these questions or skip this entire section and go on to question 47 on page 9.

About how <u>often</u> did your child…

	Never	Rarely	Sometimes	Very often	Extremely often
a. …talk?	○	○	○	○	○
b. …say his or her name when asked?	○	○	○	○	○
c. …respond when spoken to, for example, did your child look at you when you called his or her name?	○	○	○	○	○
d. …speak in sentences of at least 3 words?	○	○	○	○	○
e. …use words to request things, for example when he or she wanted a cookie?	○	○	○	○	○

41. Still remembering when your child with Fragile X Syndrome was 3 years old, about how <u>often</u> did your child…

	Never	Rarely	Sometimes	Very often	Extremely often
a. …correctly identify people or objects when you pointed to them and asked what they were, for example, “mommy”, “daddy”, “dog”, or “airplane”?	○	○	○	○	○
b. …follow simple directions, for example “sit down” or “get your shoes”?	○	○	○	○	○
c. …point at things to request them, for example, a toy on a shelf?	○	○	○	○	○
d. …copy others, for example, clapping their hands or waving?	○	○	○	○	○
e. …play pretend, for example, rocking a doll to sleep or feeding a stuffed animal?	○	○	○	○	○
f. …have savant ability, a restricted skill superior to their age group, for example reading early, or memorizing books?	○	○	○	○	○

42. If you indicated your child with Fragile X Syndrome has or had a savant ability, what is it?

43. Still remembering when your child with Fragile X Syndrome was 3 years old, how often did your child…

	Never	Rarely	Sometimes	Very often	Extremely often
a. …like motion activities, for example, to be swung or bounced?	◯	◯	◯	◯	◯
b. …walk?	◯	◯	◯	◯	◯
c. …toe walk?	◯	◯	◯	◯	◯
d. …pick up small objects, for example, Cheerios?	◯	◯	◯	◯	◯
e. …feed him or herself with a spoon?	◯	◯	◯	◯	◯
f. …help dress him or herself, for example pull up their pants?	◯	◯	◯	◯	◯

44. Still remembering when your child with Fragile X Syndrome was 3 years old, how often did your child…

	Never	Rarely	Sometimes	Very often	Extremely often
a. …get upset by loud noises, for example, the vacuum cleaner or microwave?	◯	◯	◯	◯	◯
b. …do rocking, hand flapping or spinning over and over again?	◯	◯	◯	◯	◯
c. …cry excessively over small hurts?	◯	◯	◯	◯	◯
d. …have temper outbursts if he or she did not get their way?	◯	◯	◯	◯	◯
e. …isolate him or herself?	◯	◯	◯	◯	◯
f. …try to injure him or herself, for example, head banging?	◯	◯	◯	◯	◯

45. Still remembering when your child with Fragile X Syndrome was 3 years old, how often did your child…

	Never	Rarely	Sometimes	Very often	Extremely often
a. …get upset by minor changes to their daily routine?	◯	◯	◯	◯	◯
b. …have difficulty expressing his or her needs and desires?	◯	◯	◯	◯	◯
c. …hate crowds, for example, difficulties in restaurants or the grocery store?	◯	◯	◯	◯	◯
d. …not like to be touched or held?	◯	◯	◯	◯	◯
e. …like to play with other children?	◯	◯	◯	◯	◯

46. Still remembering back to when your child with Fragile X Syndrome was 3 years old, how often did you think your child…

	Never	Rarely	Sometimes	Very often	Extremely often
a. …had an anxiety problem?	○	○	○	○	○
b. …had a hearing problem?	○	○	○	○	○
c. …had a vision problem?	○	○	○	○	○
d. …had a learning problem?	○	○	○	○	○

47. What is your relationship to the child with Fragile X Syndrome?

○ Mother
○ Father
○ Sister
○ Brother
○ Grandparent
○ Caretaker
○ Teacher
○ Other relationship → Please tell us: ______

48. What is the sex of your child with Fragile X Syndrome?

○ Female
○ Male

49. Which of the following describe your child's race or ethnicity? Please check all that apply

☐ American Indian or Alaskan Native
☐ Asian
☐ Black or African American
☐ Hispanic or Latino
☐ Native Hawaiian or Other Pacific Islander
☐ White
☐ Other race or ethnicity: Please tell us: → ______

50. What is your child's date of birth?

MM DD YYYY

51. What was your child's length in inches at birth? If you are not sure, please provide your best estimate.

______ Inches

52. What was your child's weight at birth? If you are not sure, please provide your best estimate.

[ ] Pounds [ ] Ounces

53. What is your child's current height? If you are not sure, please provide your best estimate.

[ ] Feet [ ] Inches

54. What is your child's current weight? If you are not sure, please provide your best estimate.

[ ] Pounds

55. What is the date on which you completed this questionnaire?

[ ] [ ] [ ]

MM DD YYYY

Thank you for agreeing to participate in our research study on Fragile X Syndrome.
We want you to know that your time and effort are greatly appreciated.
